# Near-Field Probing of Optical Superchirality with Plasmonic Circularly Polarized Luminescence for Enhanced Bio-Detection

**DOI:** 10.1021/acsphotonics.2c01073

**Published:** 2022-10-20

**Authors:** Victor Tabouillot, Rahul Kumar, Paula L. Lalaguna, Maryam Hajji, Rebecca Clarke, Affar S. Karimullah, Andrew R. Thomson, Andrew Sutherland, Nikolaj Gadegaard, Shun Hashiyada, Malcolm Kadodwala

**Affiliations:** † School of Chemistry, Joseph Black Building, University of Glasgow, GlasgowG12 8QQ, U.K.; ‡ School of Engineering, Rankine Building, University of Glasgow, GlasgowG12 8LT, U.K.; § Department of Electrical, Electronic, and Communication Engineering, Chuo University, 1-13-27 Kasuga, Bunkyo-Ku, Tokyo112-8551, Japan

**Keywords:** plasmonic, superchirality, chirality, near field

## Abstract

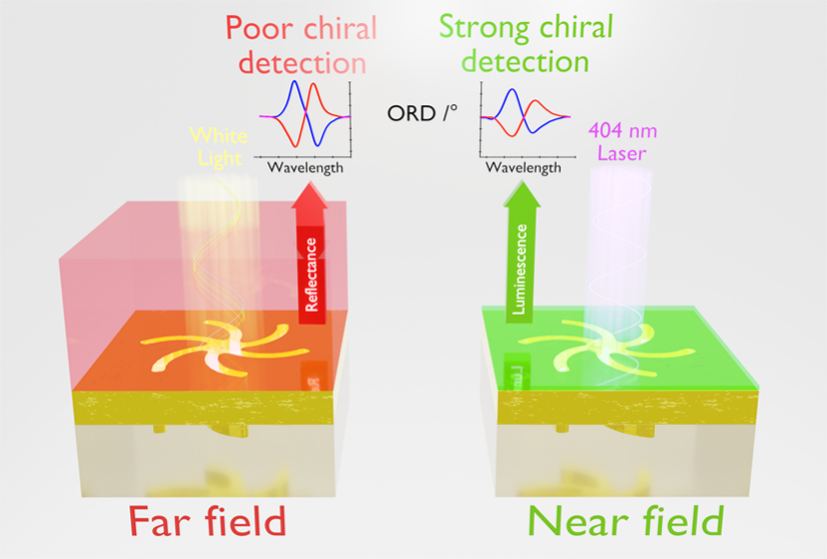

Nanophotonic platforms in theory uniquely enable < femtomoles of chiral biological and pharmaceutical molecules to be detected, through the highly localized changes in the chiral asymmetries of the near fields that they induce. However, current chiral nanophotonic based strategies are intrinsically limited because they rely on far field optical measurements that are sensitive to a much larger near field volume, than that influenced by the chiral molecules. Consequently, they depend on detecting small changes in far field optical response restricting detection sensitivities. Here, we exploit an intriguing phenomenon, plasmonic circularly polarized luminescence (PCPL), which is an incisive local probe of near field chirality. This allows the chiral detection of monolayer quantities of a *de novo* designed peptide, which is not achieved with a far field response. Our work demonstrates that by leveraging the capabilities of nanophotonic platforms with the near field sensitivity of PCPL, optimal biomolecular detection performance can be achieved, opening new avenues for nanometrology.

## Introduction

Chiral pharmaceutical and biological molecules are detected and characterized using chirally sensitive spectroscopic techniques based on the differential absorption and scattering of circularly polarized light (CPL). This absorption asymmetry between left-handed (LH) and right-handed (RH) CPL of a chiral molecule is dictated by a property known as optical activity,^1^ which will manifest with two optical responses, optical rotation and circular dichroism (CD).^2^ These effects are measurable by shining linearly polarized light (as the sum of left- and right-CPL) through optically active media and characterizing the polarization state of the reflected or transmitted light. Chiral molecules often show a small CD response in the order of tens of millidegrees for an ultraviolet incident beam of light.^3^ Potentially, chirally sensitive (chiroptical) spectroscopy could have a broad impact; however, the inherent weakness of the dichroic interactions intrinsically limits its applications.

The sensitivity of chiral spectroscopic measurements can be vastly improved by leveraging the capabilities of nanophotonics to enhance chiral light matter interaction and thus make the chiral responses larger. The chiral light–matter interactions can be amplified by using artificial chiral nanostructures because of their strong optical activities over a large range of frequency (visible to infrared).^4,5^ These plasmonic nanostructures exhibit near fields with both enhanced intensities and chiral asymmetries. This property is sometimes referred to as superchirality.^6−8^

Using pairs of enantiomorphic plasmonic chiral nanostructures, near fields of opposite symmetry can be produced, which interact asymmetrically with chiral media. This asymmetry causes differential changes in both the intensities and chiral asymmetries of the near fields in the vicinity of the chiral media. These local changes in near field properties are then detected through classical light scattering or absorption far field measurements. The sensitivity of these types of measurements, like all plasmonic-based sensing strategies, is limited by the volume occupied by the molecules of interest (which are typically monolayers adsorbed on to the surface of nanostructures) relative to the spatial extent of the near fields.^9^ Consequently, plasmonic-based sensors are less sensitive to relatively small molecules which occupy a smaller fraction of the near field environment. This intrinsically limits the sensitivity and applicability of plasmonic-based sensing techniques.

We report a novel phenomenon which enhances the sensitivity of plasmonic-based chiral detection. Specifically, we demonstrate that plasmonic circularly polarized luminescence (PCPL) is far more sensitive to local changes in the optical chirality of the near field than far field light scattering-based measurements. Thus, a small chiral molecule can be detected, which is otherwise undetectable by reflectance-based light scattering measurements.

In general, luminescence is used in two distinct ways in chiroptical spectroscopic measurements. The total yield of luminescence can be used to monitor differential absorption in CD measurements for biomolecules which contain luminescent residues. Alternatively, circularly polarized luminescence can be used to monitor the chirality of luminescent materials. In the case of organic (bio)molecules, the asymmetries observed in circularly polarized luminescence are very small ≪ 0.1%; this combined with the requirement of luminescent residues limits the potential impact of the technique.^10^ The PCPL phenomenon does not suffer from these limitations; it does not require the (bio)molecule to have significant luminescence, instead it relies on monitoring changes in large dichroic signals (asymmetries of the order of tens of percent) from chiral plasmonic structures.

We attribute the enhanced sensitivity of PCPL to the fact that the signal is dependent on the local electromagnetic (EM) environment in the vicinity of the surface, the region occupied by adsorbed chiral molecules, rather than the significantly larger volume of the overall near field from which the far field response is derived. The ability of PCPL to provide greater sensitivities widens the potential application of chiral metamaterials for bio/enantiomeric detection.

It has been known for over 50 years that illuminating gold films with ultraviolet light results in photoluminescence which spans the visible to near-infrared region.^11^ This light emission is due to the direct radiative recombination of electrons near the Fermi level with holes in the d-band. Early work also demonstrated that the amount of luminescence was enhanced by local fields generated by plasmonic excitation.^12^ The basis of the PCPL strategy is that the polarization properties of the luminescence are governed by the chiral asymmetry of the EM near fields in the vicinity of the gold surface. The veracity of this assumption is provided by work which has demonstrated that achiral dye molecules embedded in a matrix surrounding a chiral plasmonic structure emitted CPL, and that the degree of circular polarization is correlated to the level of chiral asymmetry of the near fields.^13^ Later work confirmed enhanced photoluminescence effects of chiral and achiral molecules in plasmonic near fields.^14,15^ The chiral asymmetries of the near fields in these previous studies, and others concerning chiral nanophotonics, have been parameterized using an optical chirality(*C*) factor.^6−8,16^ The time average value of *C* is defined by:
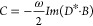
1

where *D* = ϵ(ω)*E* and *B* = μ(ω)*H* with ϵ = ϵ′ + *i*ϵ″ and μ = μ′ + *i*μ″ are the complex electric permittivity and magnetic permeability, respectively; *E* and *H* are complex time harmonic electric and magnetic fields, and ω is the angular frequency. The *C* factor is a conserved quantity of an EM field^17^ and provides a convenient quantity for parameterizing the chiral asymmetries of EM fields.^16^ In more recent work,^18^ it has been reported that the optical chirality flux (*F*), which by analogy with Poynting’s theorem is identified in a conservation law for *C*, is proportional to the degree of circular polarization. ***F*** is defined as:
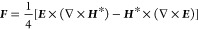
2

In subsequent discussions of the PCPL, the observed levels of asymmetries are rationalized in terms of *F_z_*, the component of the optical chirality flux parallel to the propagation of the incident beam that is being measured experimentally. In current work, we have normalized both *C* and *F_z_* to the values for right-CPL.

## Experimental Section (Materials and Methods)

### Sample Nanofabrication

Polycarbonate slides having shuriken nanoindentations were manufactured using an injection molding machine (ENGEL); the procedure is described in detail elsewhere.^19^ The shuriken indentation on the substrate had a depth of ∼80 nm and a length of 500 nm from arm to arm. After fabrication, the slide was cleaned with isopropyl alcohol and dried with N_2_ gas. The cleaned slide was metal evaporated using a Plassys MEB-400s to a gold thickness of 100 nm and then cleaned in an oxygen plasma asher (see the Supplementary Information).

### *cc-Hept* Functionalization

The *cc-Hept* monolayer was deposited on the gold substrate after conducting all the measurements in phosphate buffer saline (PBS). Oxygen plasma treatment was conducted for 1 min at 80 W before placing the sample in a 0.1 mM solution of *cc-Hept* diluted in HEPES buffered saline (HBS, 10 mM HEPES and 150 mM NaCl in water at pH 7.2). After 24 h of incubation, the sample was rinsed using HBS to flush out the unbound molecules.

### Far Field Optical Measurement Setup

**Figure 1:**
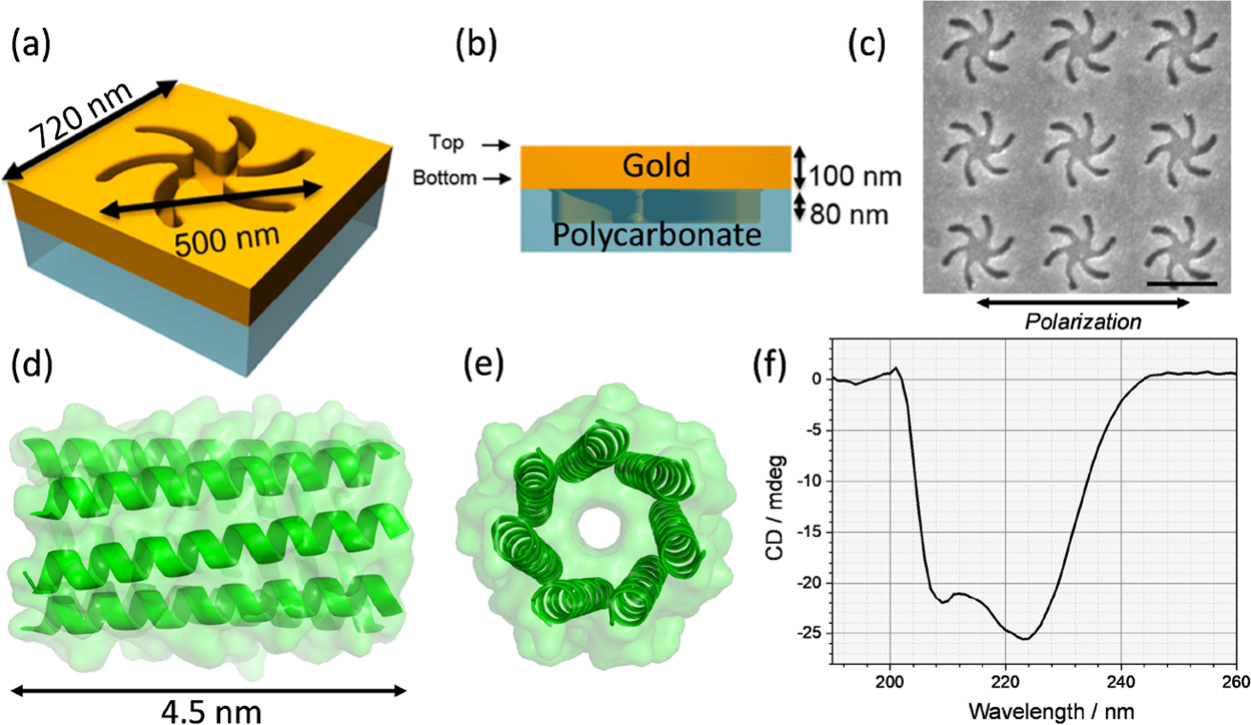
Schematic of the (a) orthographic and (b) side view of the (LH) TPS metafilm. (c) Scanning electron microscopy (SEM) image of a LH TPS with the scale bar showing 500 nm. (d) Side view of the structure of the *cc-Hept* peptide and (e) top view. (f) CD spectrum of 50 mM *cc-Hept* in HEPES buffer.

**Figure 2:**
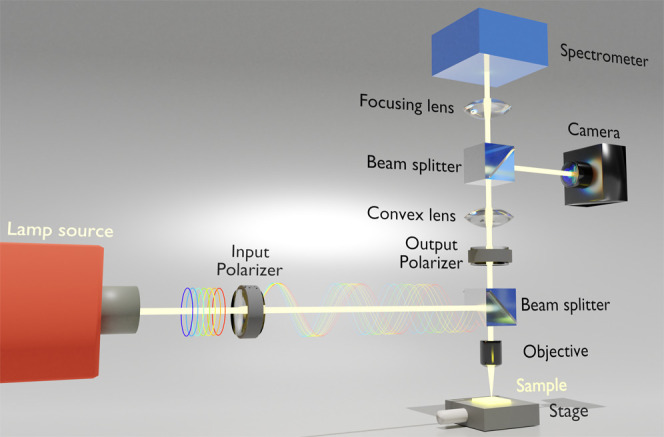
Polarization microscope utilized to measure the far field ORD.

Spectra were recorded from enantiomorphic pairs of template plasmonic substrates (TPS) while they were immersed in buffer PBS (pH of 7.4). Measurements were made on substrates which were either unfunctionalized or functionalized with *cc-Hept*. To provide achiral reference datasets for comparison we have also collected data from salt solutions. The far field chiroptical response was obtained using optical rotatory dispersion (ORD) which monitors the level of optical rotation (θ) as a function of wavelength. ORD measurements of our samples were performed with a custom-made polarization microscope as described previously in Figure 2. The instrument consists of a tungsten halogen lamp (Thorlabs) light source that propagates through a collimating lens, Glan–Thomson polarizer (Thorlabs), 50:50 beamsplitter, and a focused beam 10× objective lens (Olympus). The reflected beam propagates back from the second Glan–Thompson polarizers (Thorlabs) and is captured by the spectrometer (Ocean Optics USB4000). The sample was positioned and focused using a camera (Thorlabs DCC1645C) after the analyzer, allowing the polarized beam to hit the nanostructures at the orientation shown in Figure 1c. The Stokes method is used to record the ORD spectra at four analyzer angles (0°, ±45° and 90°) concerning the incident polarization for enantiomorphic pairs of TPSs (see the Supplementary Information).

### Near Field Photoluminescence Setup

Near field measurements were obtained from the same substrates as those used to acquire the far field data. In contrast to the far field measurements, both θ and ϕ spectra were collected. The measurements were conducted using a home-built microscope from Thorlabs (ESI) depicted in Figure 4. A 404 nm laser diode, with a 180 mA fixed current and maximum optical power output of ∼17 mW, was used as the excitation source. Two linear polarizers at the input after the laser diode were mounted to adjust the input power. The first polarizer was used to modify the input power and the second polarized to define the input polarization. The beam was focused using a 10× (NA = 0.3) objective and the photoluminescence signal was collected with the same objective in a reflection geometry. The position of the sample and alignment of the laser were monitored using an optical camera. The sample was positioned such that the polarized beam was at the same orientation as for the far field measurements, as shown in Figure 1c. A charged-coupled device camera mounted on the top of the configuration was used to capture the signals (see the Supplementary Information).

### Numerical EM Modeling

A commercial finite element package (COMSOL V5.6 Multiphysics software, Wave Optics module) has been used to simulate the intensity and chiral asymmetry of EM fields produced across the sample. Periodic boundary conditions have been imposed on the sides of the metafilm (i.e., equivalent to simulating a metafilm array). Reflections were minimized using a perfectly matched layer above and below the input and output ports.

Linearly polarized EM waves were focused at normal incidence onto the metafilm. The finite-element method has been used to solve Maxwell’s equations over a distinctive geometry which allows the optical chirality and *E*-field intensity to be measured in COMSOL. A 10 nm continuous dielectric domain, with a refractive index of 1.44, has been defined at the surface of the sample to simulate the *cc-Hept* monolayer. Above this layer, a 540 nm domain containing water (*n* = 1.331) has been defined on top of the chiral layer, allowing the calculation of the far field volume average of *F_z_* (see the Supplementary Information).

## Results and Discussion

The samples used in this study are made of a ∼100 nm thick gold metafilm deposited on a nanostructured polycarbonate template.^20^ The polycarbonate platform consisted of either LH or RH “shuriken” shaped indentations, Figure 1a–c, possessing six-fold rotational symmetry, that were arranged in a square lattice. These substrates are referred to as TPS. The nanoscale indentations in the surface polycarbonate substrate have a depth of ∼80 nm, are 500 nm in diameter from arm-to-arm, and have a pitch of 720 nm. A detailed discussion of the chiral and optical properties of these substrates can be found elsewhere.^21^

As a well-defined, relatively small model biological molecule, we used a synthetic *de novo* designed heptameric α-helical barrel assembly. Seven identical peptide chains self-assembled into a higher order α-helical coiled coil. The resultant barrel structure is stable to chemical and thermal denaturation. To enable the peptide to be immobilized with a well-defined geometry on the surface, a thiol polyethylene glycol linker is incorporated into the structure, Figure 1d,e. For brevity, this coiled coil heptamer molecule will be subsequently referred to as *cc-Hept*. The CD spectrum of *cc-Hept* in buffer, Figure 1f, is qualitatively similar to that reported for a related α-helical heptamer.^22^ Based on its molecular structure, the *cc-Hept* peptide has an approximate length of 4.5 nm. However, after immobilization on the gold surface, the height of the molecular layer has been measured by ellipsometry to be 3.44 ± 0.01 nm. To assess the uniformity of the peptide layer, we have collected atomic force microscopy images. These show a uniform coverage (i.e., an absence of holes in the layer) with an observed root-mean-square roughness of 1.4 nm, a value similar to that of the unfunctionalized gold film (see Supplementary Information). The discrepancy between the predicted length of the molecule and the thickness of the immobilized layer implies that the orientation of the *cc-Hept* peptides is inclined to the surface normal.

Central to this study is the potential for differential changes in chiroptical spectra, both scattered light (far field) and luminescence (near field) from LH and RH TPS induced by the presence of adsorbed chiral media. The *A* factor, derived from the relative changes in the peak-to-peak height of resonances, is used to parameterize the asymmetries in both optical rotation and ellipticity spectra, and is defined by the following equation:
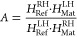
3where *H*_Ref_^RH/LH^ are the peak-to-peak heights between the minima and maxima of the line shape. The position of the minima and maxima from which the peak-to-peak heights are derived, are for ϕ (θ) ∼ 700 and 720 nm (720 and 747 nm), respectively. This is illustrated in Figures 5a–c and 6a–c, for both ϕ and θ spectra for the buffer references for RH and LH structures, respectively. *H*_Mat_^RH/LH^ are the equivalent values for the achiral salt or the *cc-Hept*. A nonunity value for this parameter indicates an asymmetry between intensities of LH and RH nanostructures’ spectra.

**Figure 3:**
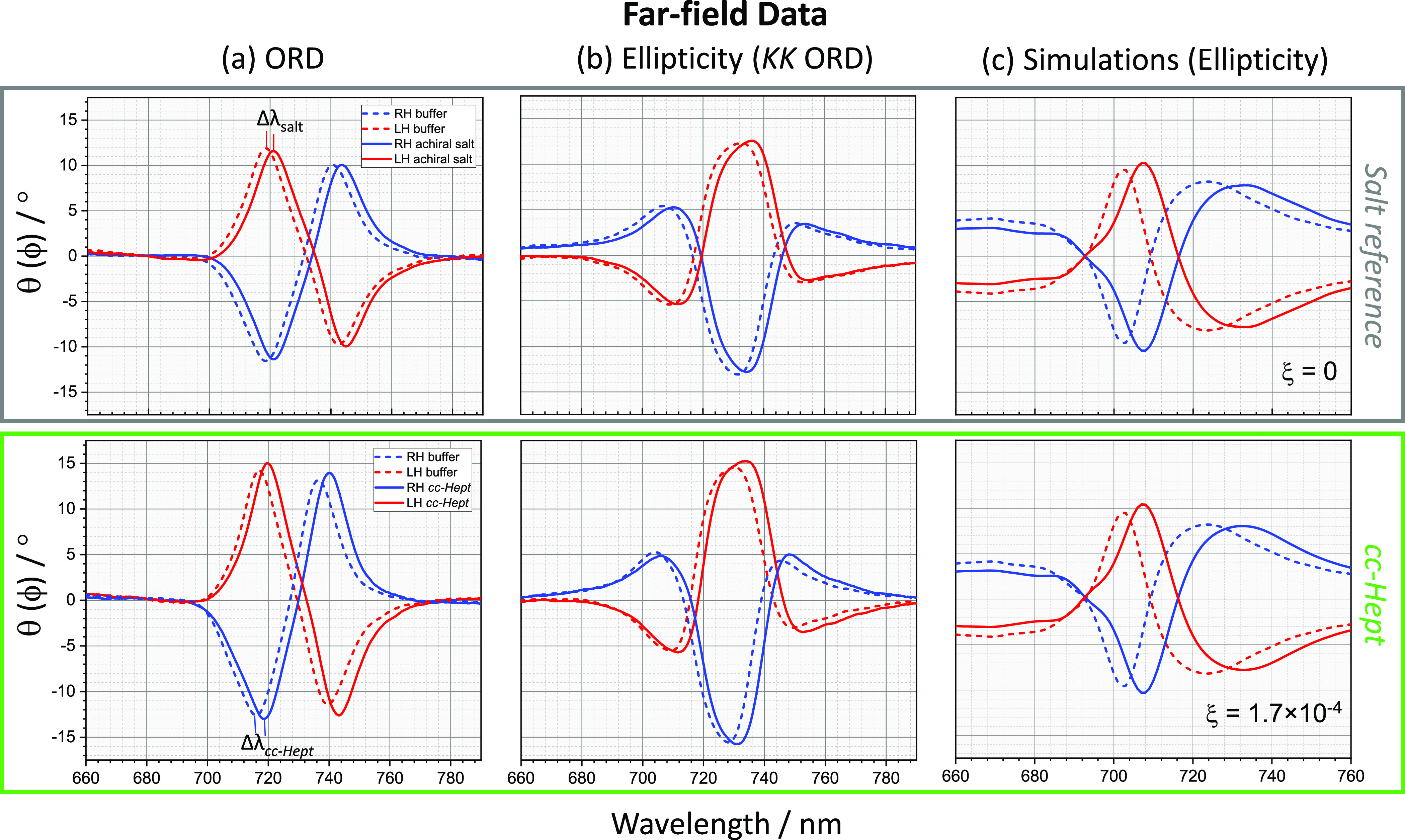
Displayed in upper and lower panels are far field data of LH (red) and RH (blue) structures, derived from light scattering measurements, for the achiral salt reference and *cc-Hept* functionalized TPS, respectively (solid line), compared to buffer (dotted line), with columns (a–c) containing ORD, ellipticity (*kk*-ORD), and simulated ellipticity spectra obtained for ξ of 0 and 1.7 × 10^–4^.

Far field ORD data were collected using a polarization microscope as shown in Figure 2. The ellipticity (ϕ) of the far field reflected beam could not be directly determined due to limitation of the used setup. However, by using the *Kramers*–*Kronig* (*KK*) relationship, the ellipticity spectra can be derived from the ORD data.^23^ Spectra from *cc-Hept* functionalized and achiral salt solutions for both LH and RH substrates are shown in Figure 3a–c, along with ellipticity spectra derived from the *KK* of the ORD. As expected, the adsorption of the *cc-Hept* causes red shifts in the positions of the resonances. There is no asymmetry observed for either the achiral salt solutions or the *cc-Hept* functionalized substrates, as shown in Table 1, and the *A* factors are close to 1.

**Table 1:** Comparison of Experimental and Simulation Asymmetry Factors for the Achiral Salt Solution and the *cc-Hept* Chiral Monolayer with the Standard Errors for Experimental Values Derived from Four Datasets

*A* (asymmetry parameter)	salt	*cc-Hept*
far field ORD (expt.)	1.00 ± 0.05	1.03 ± 0.05
far field ORD (sim.)	1.00	1.00
near field ORD (expt.)	1.00 ± 0.07	1.25 ± 0.07
near field ORD (sim.)	1.00	1.25
near field ellipticity (expt.)	0.98 ± 0.07	1.25 ± 0.07
near field ellipticity (sim.)	1.00	1.25

**Figure 4:**
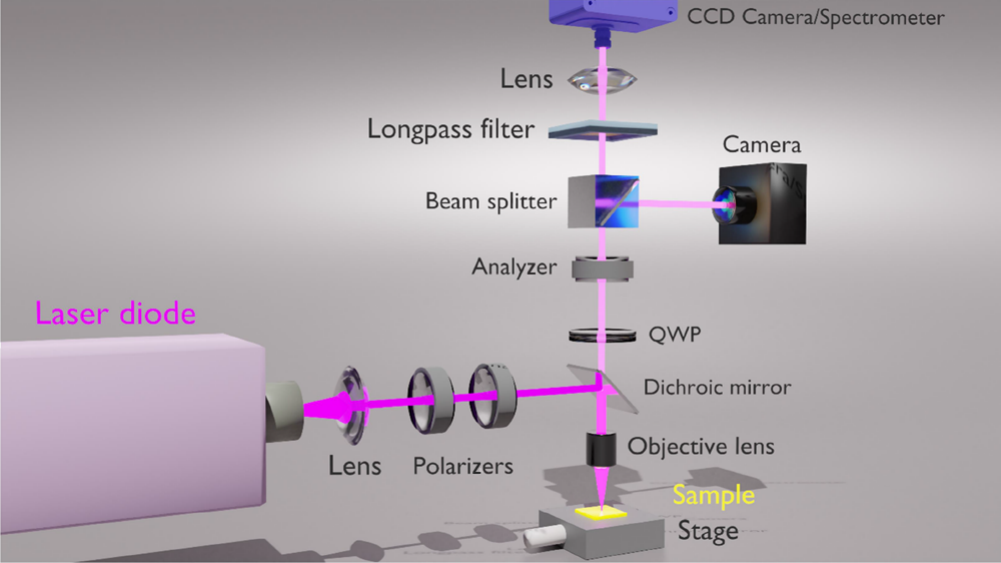
Photoluminescence setup used to obtain the near field ORD and ellipticity.

**Figure 5:**
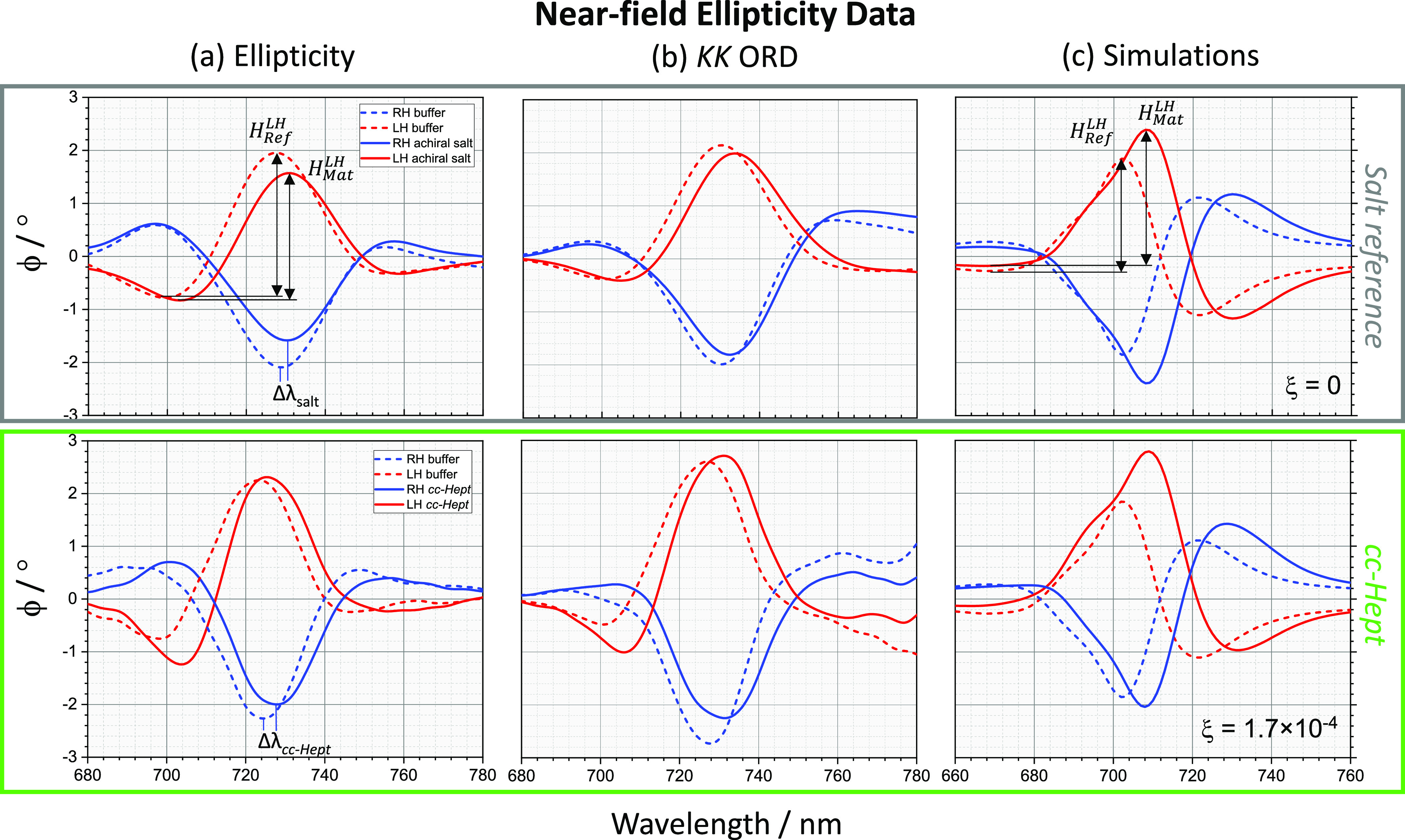
Displayed in upper and lower panels are near-field ellipticity data of LH (red) and RH (blue) structures, for the achiral salt reference and *cc-Hept* functionalized TPS, respectively (solid line), compared to buffer (dotted line), with columns (a–c) containing ellipticity, ellipticity derived from *kk*-ORD, and simulated ellipticity spectra obtained for ξ of 0 and 1.7 × 10^–4^. In the upper panels (a,c), the asymmetry parameter *A* for ellipticity data is illustrated.

**Figure 6:**
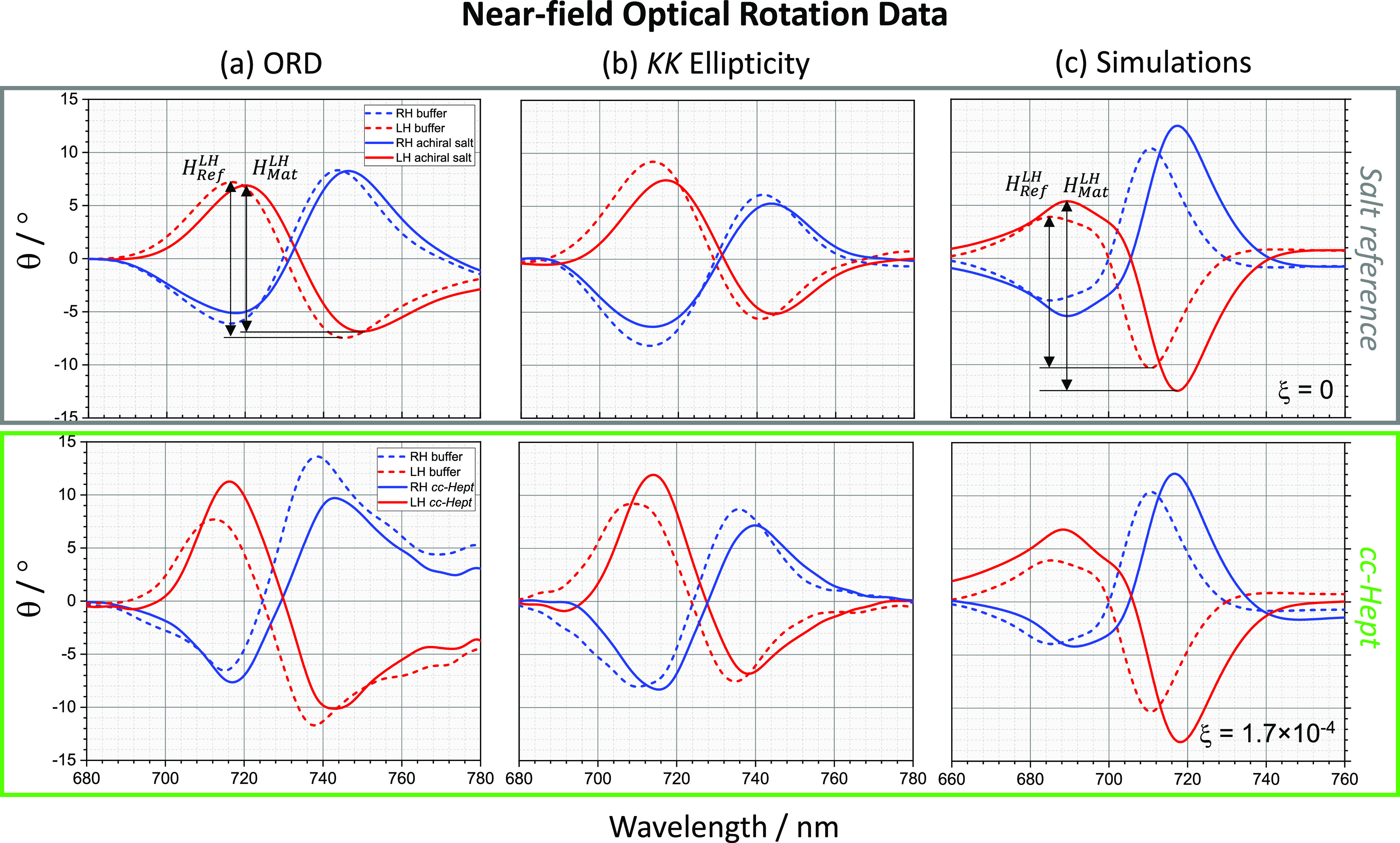
Displayed in upper and lower panels are near-field optical rotation data of LH (red) and RH (blue) structures, for the achiral salt reference and *cc-Hept* functionalized TPS, respectively (solid line), compared to buffer (dotted line), with columns (a–c) containing optical rotation, optical rotation derived from *kk*-ellipticity, and simulated optical rotation spectra obtained for ξ of 0 and 1.7 × 10^–4^. In the upper panels (a,c), the asymmetry parameter *A* for optical rotation data is illustrated.

The luminescence-derived ϕ and θ spectra for both the achiral salt solutions and *cc-Hept* functionalized substrates were measured using the setup shown in Figure 4 and are shown in Figures 5a and 6a, with asymmetry factors given in Table 1. The similarity between the experimental and the corresponding *KK* data demonstrates the robustness of the measurements. The *cc-Hept* data exhibit significant asymmetries, while no discernible asymmetries are observed for the achiral salt solution data.

The asymmetry factor *A* had been used to parameterize spectral asymmetries previously,^24^ and it was found that *A* depends on the net charge distribution and the chirality of the biomolecule. It was observed that *A* has a value greater than 1 if the biomolecule has a net positive charge, less than 1 for a net negative charge, and 1 in the case of no net charge. The net charge on a biomolecule is determined by its isoelectric point (IP) and the pH of the surrounding buffer. If the IP is close to the pH of the buffer, there is no net charge, while at pH lower/greater than the IP, the biomolecules have a net positive/negative charge. In the case of the *cc-Hept* peptide, with an IP = 10 [23] and a buffer pH of 7.2, a net positive charge would be expected. This is consistent with the observed *A* of 1.25 ± 0.07 (see Table 1) measured here. For comparison, proteins studied in ref (23), which had net positive charges, gave *A* values in the range of 1.15 to 1.25.

To validate the veracity of the light scattering and luminescence measurements, we have performed EM numerical simulations. The modeling uses an idealized shuriken structure, and the *cc-Hept* layer is mimicked by a 10 nm thick isotropic chiral layer. Due to computational constrains, specifically the size and number of mesh elements, the minimum thickness of the chiral layer that we could accurately simulate was 10 nm. The EM simulations are based on the implementation of the following constitutive chiral relationships:^25,26^
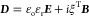
4
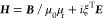
5

**Figure 7:**
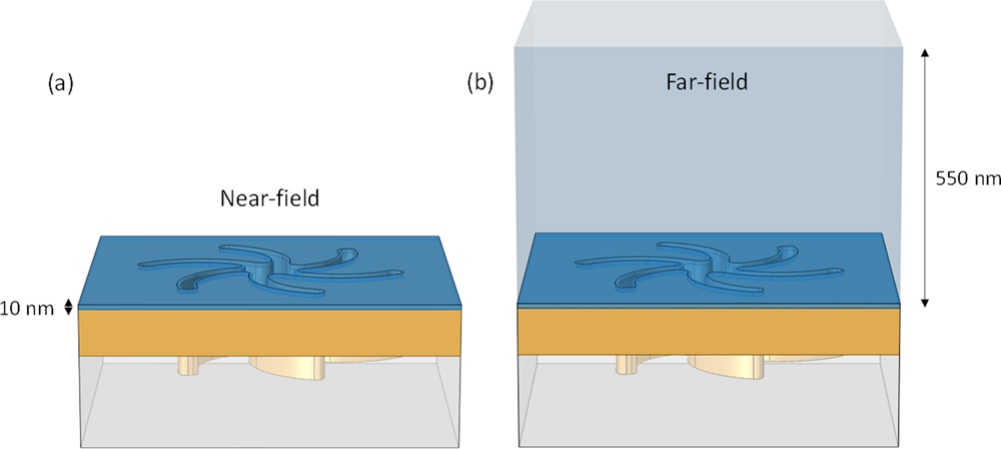
Representation of the COMSOL model used to simulate the sample responses, with (a) the 10 nm near-field chiral layer representing the *cc-Hept* monolayer at the gold surface, and (b) the far field layer comprising the chiral layer plus 540 nm of water.

Here, ε_o_ is the permittivity of free space, ε_r_ is the relative permittivity, μ_0_ is the permeability of free space, μ_r_ is the relative permeability, ***E*** is the complex electric field, ***B*** is the complex magnetic flux density, ***H*** is the magnetic field, and ***D*** is the electric displacement field. The chiral asymmetry parameter ξ^T^ is a second rank tensor describing the chiral property of a medium and is therefore zero for achiral materials. For an isotropic chiral layer, ξ_*xx*_ = ξ_*yy*_ = ξ_*zz*_ ≠ 0, while all other elements are considered zero.^21,27^ In the current simulations, we have used a ξ_*xx*_ = ξ_*yy*_ = ξ_*zz*_ = 1.7 × 10^–4^ which is consistent with values used in numerical simulations in previous work to replicate the effects of protein layers.^21^ The numerical simulations have been used to calculate volume integral values of *F_z_* in two regions of space above the surface of the TPS, Figure 7a–b, within the 10 nm thick chiral layer and the entire volume above the surface of the TPS including the chiral layer. The *F_z_* values were normalized by that for left CPL, producing a number which is equivalent to the degree of circular polarization, which can then be converted into ϕ spectra. Afterward, equivalent θ spectra can be obtained from *KK* transformation. These simulated spectra replicate the experimental luminescence and lights scattering experiments. This is illustrated by the good agreement between the *A* values obtained from the simulated spectra and those derived from the experiment, Table 1.

**Figure 8:**
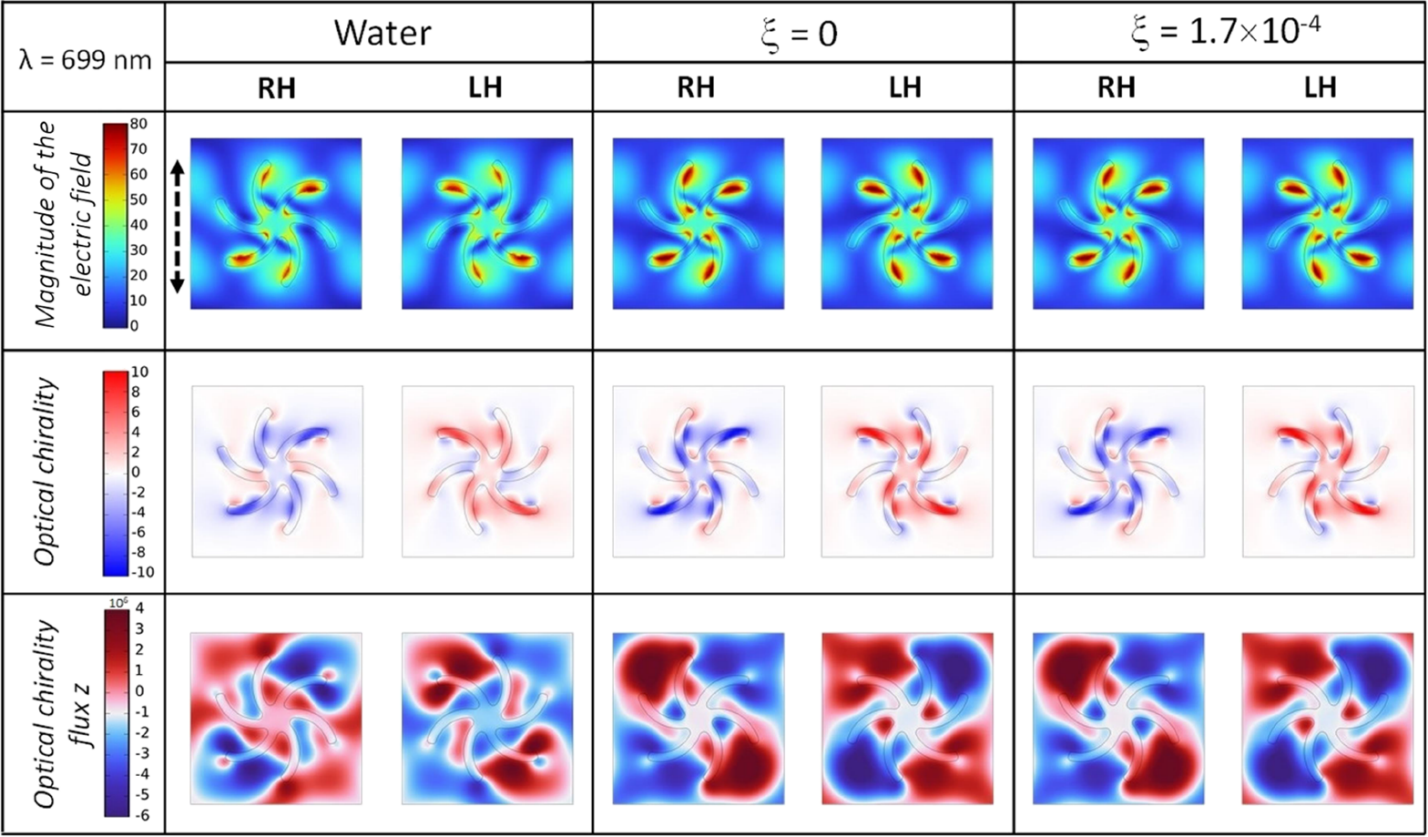
Simulation plots at 10 nm above the gold surface of the magnitude of the electric field (|*E*|), the optical chirality factor, and the optical chirality flux along the *z* axis at 699 nm for both shuriken handedness in water and with an adsorbed layer of achiral and chiral material with ξ values equal to 0 and 1.7 × 10^–4^, respectively, and the same refractive index of 1.44. Polarization of the excitation beam is represented by the dashed arrow.

Maps showing the spatial distribution of the electric field norm, the optical chirality factor *C*, and the optical chirality flux *F_z_* generated by incident light linearly polarized at 699 nm are shown in Figure 8. As expected, the plots for the water and achiral layer have a symmetrical distribution for both handedness. However, when the chiral dielectric layer is introduced in the model, the symmetry of the field is broken. The average values of the field, *C*, and *F_z_* in the near field and far field volumes are displayed in Table 2, for water, achiral, and chiral adsorbed dielectric layers. The asymmetry in both volume averaged *C* and *F_z_* of the near fields is significantly greater than that in the far field for the chiral dielectric simulation, which is consistent with experimental results.

**Table 2:** Comparison of the Volume Averaged Values of the Magnitude of the Electric Field (|*E*|), Optical Chirality Factor, and Optical Chirality Flux along the *z* Axis (Normalized to RCPL) in the Near Field and Far Field Volumes of the Simulations^a^

volume averaged	shuriken handed ness	water			ξ = 0			ξ = 1.7 × 10^–4^		
		|*E*|	*C*	*F_z_*	|*E*|	*C*	*F_z_*	|*E*|	*C*	*F_z_*
far field	RH	12.53	–0.16	–0.08	12.41	–0.22	–0.03	12.44	–0.21	–0.04
	LH	12.53	0.16	0.08	12.41	0.22	0.03	12.39	0.23	0.03
near field	RH	24.23	–0.42	–0.16	22.26	–0.39	–0.12	22.32	–0.30	–0.15
	LH	24.23	0.42	0.16	22.26	0.39	0.12	22.18	0.49	0.08

a These values were obtained for an incident beam wavelength of 699 nm.

## Conclusions

We have shown that chiroptical measurements based on luminescence from plasmonic metafilms can detect a chiral response from a *de novo* designed peptide which is undetectable with classical light scattering based measurements. The enhanced sensitivity of luminescence measurements is attributed to the fact that they are more sensitive to the surface region, which is occupied by the adsorbed chiral molecule, than the far field light scattering data. This hypothesis is supported by numerical simulations which replicate the experimentally observed behavior. By benchmarking PCPL against far field light scattering measurements from the same sample, we illustrate the enhanced sensitivity of luminescence based optically active probes for bio/enantiomeric detection. The enhanced chiral sensing capabilities of PCPL compared to far field scattering are also apparent when the current study is compared to previous work. Many of these previous studies, which involved detecting biomolecules (proteins and viruses) of significantly greater molecular mass than the peptide studied here, have focused on measuring asymmetric shifts in the positions of plasmonic resonances. The typical asymmetries measured in these studies are at the level of 30% of the average shift. Consequently, this strategy is only viable for relatively large biomolecules (polymeric proteins and viruses,^28,29^ that have masses >10 times greater than the peptide studied here) which produce significant shifts (>5 nm), and thus measurable asymmetries. For monolayers of relatively small biomolecules, such as the peptide studied here, which produced an average shift of ∼2 nm (relative to buffer), the predicted asymmetries would be approaching the detection limit of the method. This new paradigm of exploiting luminescence as a local probe of changes in near field optical chirality induced by a chiral material has potential applications in nanometrology and point-of-care diagnostics.

## Supporting Information Available

Details on sample fabrication; further experimental information; EM simulations (PDF)
