# Supplementary material for: Near-Field Probing of Optical Superchirality with Plasmonic Circularly Polarized Luminescence for Enhanced Bio-Detection
Source: ACS Photonics. 2022 Oct 20;9(11):3617–24. doi: 10.1021/acsphotonics.2c01073 (PMC9673156; doi:10.1021/acsphotonics.2c01073)
Supplement: Supplementary file 1 — ph2c01073_si_001.pdf [file ph2c01073_si_001.pdf]

Supporting Information

Near field Probing of Optical Superchirality with Plasmonic Circularly Polarized Luminescence for Enhanced Bio-detection

*Victor Tabouillot[1] *, Rahul Kumar[1], Paula L. Lalaguna[1], Maryam Hajji[1], Rebecca Clarke[1], Affar S. Karimullah[1], Andrew R. Thomson[1], Andrew Sutherland[1], Nikolaj Gadegaard[2], Shun Hashiyada[3] and Malcolm Kadodwala[1] **

[1] School of Chemistry, Joseph Black Building, University of Glasgow, Glasgow, G12 8QQ, UK

[2] School of Engineering, Rankine Building, University of Glasgow, Glasgow G12 8LT, UK

[3] Department of Electrical, Electronic, and Communication Engineering, Chuo University, 1-13-27 Kasuga, Bunkyo-Ku, Tokyo 112-8551, Japan.

Sample fabrication

Templated Plasmonic Substrates (TPS):

The TPSs are made using and an injection moulding machine (ENGEL), following the technique explained by Gadegaard et al. [1]. The master shim for this is made using e-beam lithography. To create the master, 100 nm of PMMA is spin coated onto a Si wafer and baked for an hour at 180 ˚C. The resist is patterned using a VB6 UHR EWF lithography tool (Vistec). The exposed resist is developed in IPA and Methyl Isobutyl Ketone, MIBK (3:1 ratio) for 60 secs. Ni is electroplated onto the surface and removed from the wafer to provide the Nickel shim that is then used as the master in a tool placed in the injection moulder. Polycarbonate pellets are thermally heated and pushed into the tool to create small plastic slides with the nanostructures indented on the surface. These slides are then coated with 100 nm of Au in an e-beam evaporator at a rate of ≈0.3 nm s-1.

The nano patterns are indentations in the surface and have a depth of ~80 nm, are 500 nm in length from arm to arm, and have a pitch of 700 nm. When gold is evaporated onto the surface, it takes the shape of the indentation and forms a hybrid plasmonic structure constituting an inverse structure at the top and a solid one at the bottom. For more information on the plasmonic behaviour of the TPS refer to Karimullah et al. [2].

Further Experimental Information

AFM imaging of the cc-Hept monolayer:

To verify the uniformity of the *cc-Hept* layer over the gold film, AFM images over large areas of the functionalized flat gold have been collected. The root mean square roughness has been found to be equal to 1.4 ± 0.1 nm over multiple areas of 3 by 3 μm, see **Figure S1**. This value is identical to the one obtained on unfunctionalized gold film in previews study [3], confirming the uniform coverage of the peptide layer.

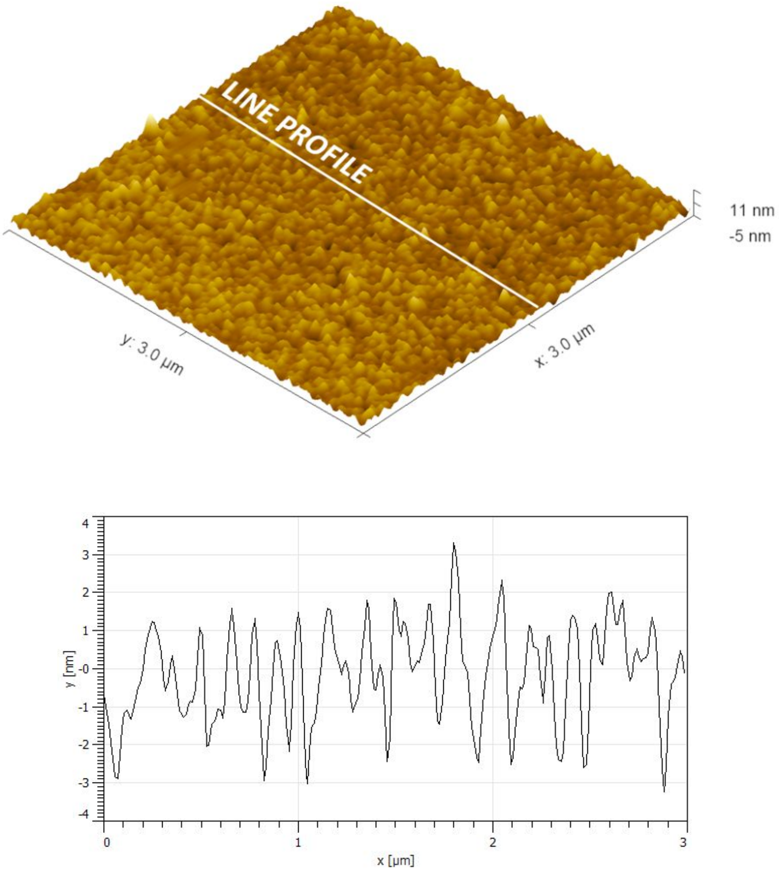

Figure S1. 3D AFM image of the *cc-Hept* functionalized gold film and (below) a line profile taken along the indicated line.

Optical measurements setup:

The far field ORD measurements were carried out using a polarisation microscope. This custom-built instrument can record ORD and reflectivity spectra. The light is generated by a 50W tungsten halogen light source from Thorlab, the beam passes through a nanoparticle polariser (Thorlab) and then sent to a beam splitter (Thorlab 50:50 700 nm-1000 nm CCM-1BSO14/M). The light is then diverted and its polarisation changes by 90°, which goes to the 10× lens with 0.3 numerical aperture Olympus UPlanFLN. The light reflected from the sample passes through the beam splitter and traverses another calcite polariser (out-put polariser). To obtain an ORD spectrum, the angle af this last polariser is set to 0°,45°,90° and 135° (Stokes angles). It is then possible to use the program LabVIEW to calculate the ORD spectrum from the intensity recorded at the Stokes angles with this formula:

$$ORD = \frac{1}{2} tan^{-1} \frac{(I_{45} - I_{135})}{(I_0 - I_{90})} \quad (0)$$

With I the intensity of the light. To record the reflectivity spectra, the out-put polariser is set to 0°.

The beam of light is passing through a second beam splitter to reach a camera (Thorlab CMOS camera DCC1645C) and the spectrometer (Ocean Optic USB 4000). Using Thorlab camera software, it is possible to focus on the nanostructures arrays and align the sample to the linearly polarised incident beam.

Far field reflectance measurements:

The reflectance of the sample was recorded for both the salt solution and the *cc-Hept* monolayer in HEPES buffer (**Figure S2**). These results were taken at the same time as the ORD measurements, simply by dividing the spectrum obtained on the nanostructures by the spectrum reflected from the gold background:

$$Reflectance = \frac{(I_{nanostructure})}{(I_{background})} \qquad (2)$$

The reflectance spectra can be influenced by the chiral medium surrounding the plasmonic nanostructures, resulting in asymmetric changes between LH and RH spectra [4]. This asymmetry is parametrized by $\Delta\Delta S$, which is the difference in separation between the two peaks of the reflectance spectra ΔS for LH and RH (see **Figure S2 (a)**):

$$\Delta S^{RH/LH} = S_{Mat}^{RH/LH} - S_{Ref}^{RH/LH} \qquad (3)$$

$$\Delta\Delta S = \Delta S_{LH} - \Delta S_{RH} \qquad (4)$$

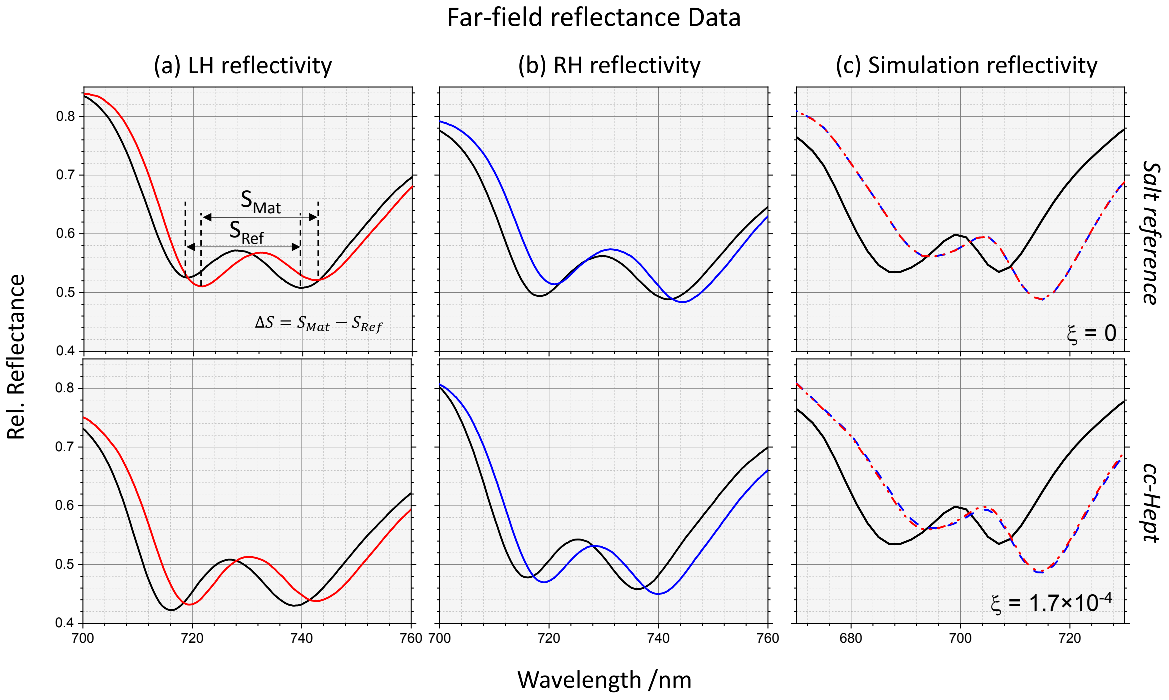

Figure S2. A Displayed in upper and lower panels are far field reflectance data for the achiral

salt reference and *cc-Hept* functionalised TPS respectively in comparison to buffer (in black), with columns (a), (b) and (c) containing reflectance for the left-handed (red), right-handed (blue) structures and the simulated reflectance spectra obtained for ξ of 0 and 1.7×10^{-4} for both handedness. In the upper panels (a) the asymmetry parameter ΔS is illustrated.

For an achiral material, $\Delta\Delta S$ is expected to be null, and when $\Delta\Delta S \neq 0$, it means that a chiral medium is present in the vicinity of the plasmonic nanostructures, interacting with the electromagnetic near field. The values of $\Delta\Delta S$ are displayed in the **Table S1**:

**ΔΔS (Asymmetry Parameter)**	***Salt***	***cc-Hept***
ΔΔS (nm) Experimental	-0.2 ± 0.3	0 ± 0.5
ΔΔS (nm) Simulation	0	1.0

Table S1. Asymmetry parameter ΔΔS compared for the achiral salt solution and the *cc-Hept* monolayer obtained experimentally (average of 4 data sets) and through EM simulation.

These results show that the far field measurements obtained through the polarisation microscope are not displaying any asymmetry.

Photoluminescence setup:

The experiments were carried out in a home-built photoluminescence setup from Thorlabs. All the components are rigidly attached with a cage-rod system. A continuous wave 404 nm laser at fixed operating current of 180 mA and a maximum output power of ~17 mW was used as

the excitation source. The power was changed during the measurement using two linear polarizers following Malus's law. The first polarizer defines the input polarization.

The linearly polarized beam was then directed towards the sample using a dichroic mirror (cut-on wavelength 505 nm) and focused onto the sample by a 10× objective (NA=0.3). The photoluminescence signal was collected in a reflection geometry through the same objective. To determine the amount of left and right CPL emitted by the sample, the beam was directed through a combination of a quarter wave plate (QWP) and linear polarizer (LP) and measurements were taken at different analyser angles with the quarter wave plate angle kept at a constant value of 0.

A 10:90 beam splitter was used to direct 10% of the signal towards a scientific camera and 90% towards the spectrometer. The laser beam was filtered out using a long-pass filter (cut-on wavelength 450 nm), placed right above the beam splitter. This allowed the laser beam to reach the optical camera, which was used to ensure that the laser was being focused on the desired position of the sample. The photoluminescence signal was detected using a CCD camera (Andor).

Photoluminescence measurements:

The measurements were done on 4 arrays of plasmonic nanostructures for both handedness. In order to obtain the optical rotation and ellipticity angle it was necessary to take the spectra for the following setup parameters: 0° QWP/ 0° LP, 0° QWP/ 45° LP, 0° QWP/ 90° LP, 0° QWP/ 135° LP, 0° QWP/ 315° LP, 45° QWP/ 45° LP, 315° QWP/ 315° LP. The resulting spectra were obtained in counts as a function of the wavelength, see **Figure S3**. The dark current baseline has been subtracted before exploiting the data.

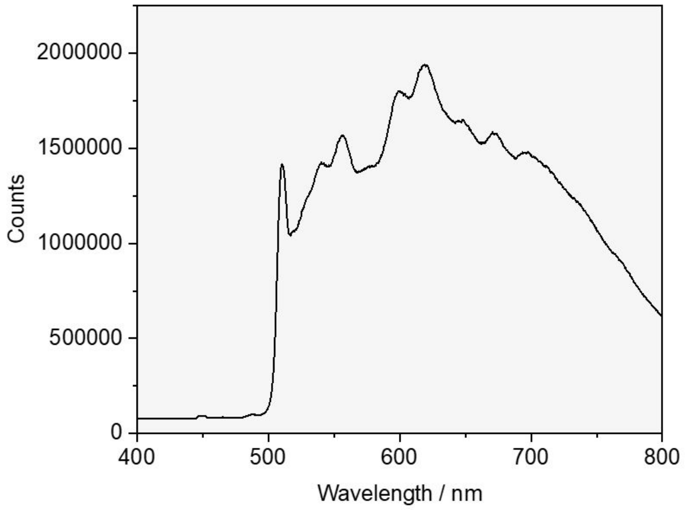

Figure S3: Photoluminescence spectrum obtained on the gold film background (without nanostructure) with the QWP and the LP set at 0°.

To ensure that the *cc-Hept* monolayer can withstand the 404 nm laser, spectra have been collected on the same nanostructures array after 12 min of total exposure time. The unsmooth ellipticity data of the LH and RH structures before and after 12 min is presented on **Figure S4**, showing no significant change in spectra, indicating that there was no radiation damage to the peptide layer over the duration of the experiment.

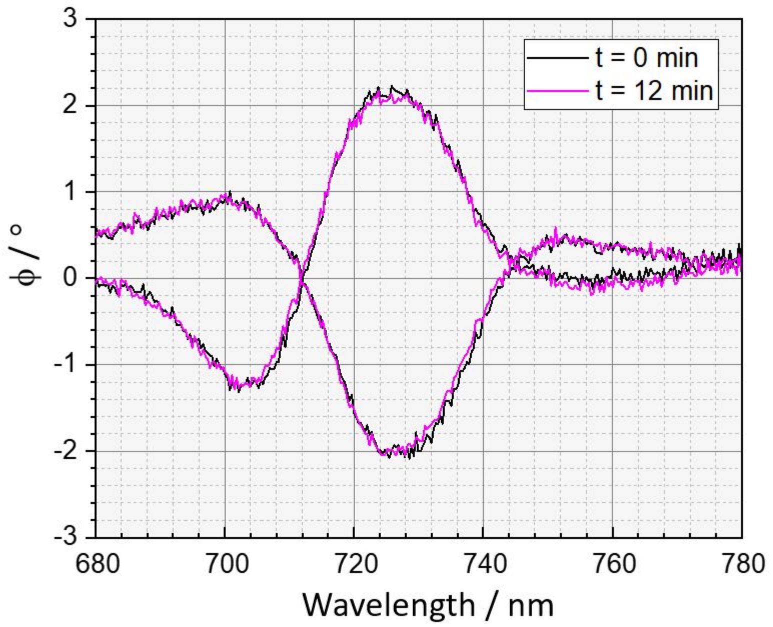

Figure S4: Unsmooth ellipticity spectra of the *cc-Hept* functionalized sample taken at 0 and 12 min of 404 nm laser illumination time for both LH and RH handedness.

Plasmonic chiral photoluminescence:

This setup can monitor both the optical rotation angle θ and the ellipticity φ, unlike the optical microscope. The angle θ is obtain in the same manner as shown in previous section with **Equation (1)**. Regarding φ, it is calculated by rotating the analyser to 45° and 315° and applying this equation:

$$\varphi = \frac{\sqrt{I_{45}} - \sqrt{I_{315}}}{\sqrt{I_{45}} + \sqrt{I_{315}}} \quad (5)$$

EM Simulation

COMSOL Model:

We have conducted a series of numerical simulations of Maxwell's equations by using finite element approach with COMSOL V6 Multiphysics software with the Wave optics module. Periodic boundary conditions are applied at the vertical boundaries of a 1600 nm heigh block of 720 by 720 nm to model the nanostructure arrays (see **Figure S5**). The idealised gold Shuriken shape of the TPS was recreated, with dimensions of 500 nm arm-to-arm, 38 nm arm width and 100 nm thick gold layer. Refractive index values for Au were taken from Johnson and Christy [5]. A 10 nm layer was added on top of the gold surface, including the side wall, to model the chiral layer of *cc-Hept* molecules. Swept mesh was used for the perfectly matched layers and tetrahedral meshing for the nanostructures and its surrounding media with automated meshing limited to a maximum element size of 18 nm in the gold and 10 nm chiral layer and 30 nm in the surrounding media. The incident light is linearly polarised along the y axis.

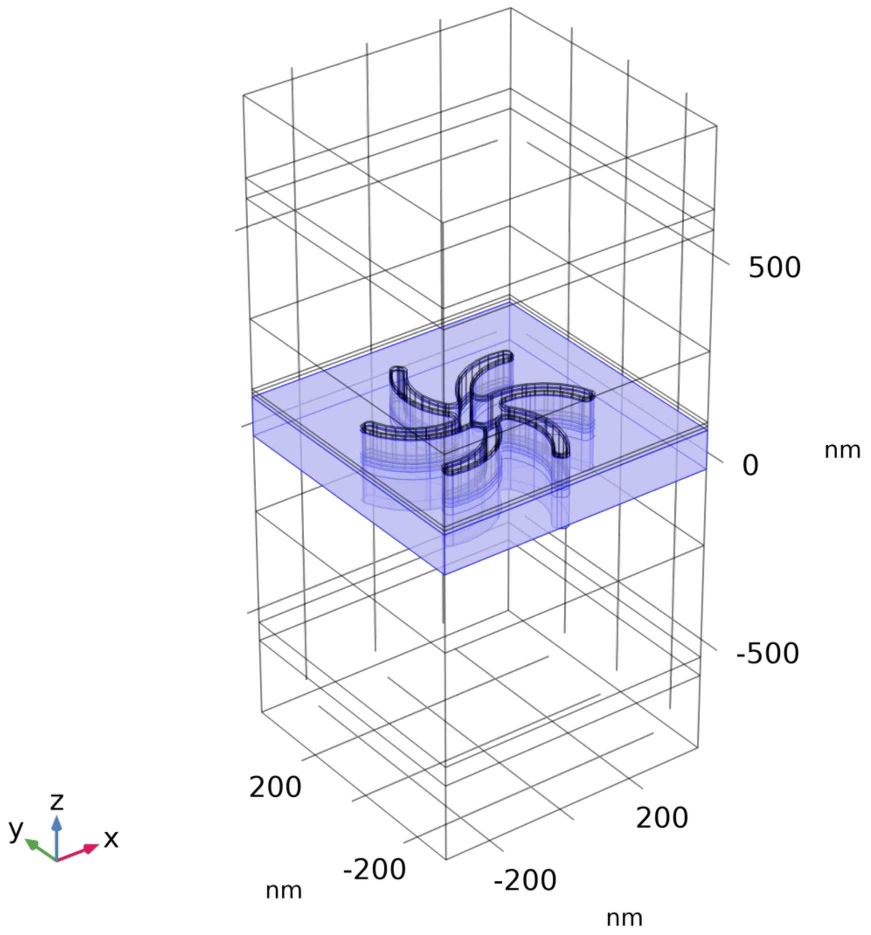

Figure S5: COMSOL model used to reproduce experimental results, with gold layer in blue.

Optical Chirality Flux volume integration:

To replicate the experimental data for the near field and far field measurements, the optical chirality flux along the z direction (F_z) has been integrated over the 10 nm chiral layer and the entire volume above the gold nanostructure respectively. The F_z equation was derived from the work of Poulikakos et al. [6]:

$$F_z = \frac{1}{4}\left[\left(E_x\left(\frac{\partial H_x}{\partial z} - \frac{\partial H_z}{\partial x}\right) - E_y\left(\frac{\partial H_z}{\partial y} - \frac{\partial H_y}{\partial z}\right)\right) - \left(H_x\left(\frac{\partial E_x}{\partial z} - \frac{\partial E_z}{\partial x}\right) - H_y\left(\frac{\partial E_z}{\partial y} - \frac{\partial E_y}{\partial z}\right)\right)\right] \quad (6)$$

References

[1] Gadegaard, N., Mosler, S. & Larsen, N. B. Biomimetic Polymer Nanostructures by Injection Molding. Macromol. Mater. Eng. 2003, **288**, 76–83.

[2] Karimullah, A. S.; Jack, C.; Tullius, R.; Rotello, V. M.; Cooke, G.; Gadegaard, N.; Barron, L. D.; Kadodwala, M. Disposable Plasmonics: Plastic Templated Plasmonic Metamaterials with Tunable Chirality. Adv. Mater. 2015, **27**, 5610–5616.

[3] MacLaren, Donald A., et al. "Asymmetric photoelectron transmission through chirally-sculpted, polycrystalline gold." Physical Chemistry Chemical Physics 11.38 (2009): 8413-8416.

[4] Tullius, Ryan, et al. "Superchiral plasmonic phase sensitivity for fingerprinting of protein interface structure." ACS nano 2017, **11.12,** 12049-12056.

[5] Johnson, P. B.; Christy, R. W. Optical Constants of the Noble Metals. Phys. Rev. B 1972, **6** (12), 4370– 4379.

[6] Poulikakos, Lisa V., et al. "Optical chirality flux as a useful far field probe of chiral near fields." ACS photonics 2016, **3.9**, 1619-1625.
